# Single minimal incision fasciotomy for chronic exertional compartment syndrome of the lower leg

**DOI:** 10.1186/s13018-016-0395-9

**Published:** 2016-05-24

**Authors:** Nicola Maffulli, Mattia Loppini, Filippo Spiezia, Alessio D’Addona, Gayle D. Maffulli

**Affiliations:** Department of Musculoskeletal Disorders, Faculty of Medicine and Surgery, University of Salerno, Salerno, Italy; Centre for Sports and Exercise Medicine, Barts and The London School of Medicine and Dentistry, Mile End Hospital, 275 Bancroft Road, London, E1 4DG England; Department of Orthopaedic and Trauma Surgery, Campus Biomedico University, Via Alvaro del Portillo, 200, 00128 Trigoria, Rome Italy; Department of Public Health, Section of Orthopaedic and Trauma Surgery, University of Naples “Federico II”, School of Medicine and Surgery, Naples, Italy

**Keywords:** Leg, Compartment syndrome, Exertional, Fasciotomy, Minimal invasive

## Abstract

**Background:**

Chronic exertional compartment syndrome (CECS) involves a painful increase in compartment pressure caused by exercise and relieved by rest, common in athletes. The most common site for CECS in the lower limbs is the anterior leg compartment. The aim of this study is to evaluate the outcomes of a single minimal incision fasciotomy in athletes and their capability to return to high level sport activity.

**Methods:**

The study reports mid-term results in a series of 18 consecutive athletes with chronic exertional compartment syndrome of the leg who had undergone minimally invasive fasciotomy. Between 2000 and 2007, we prospectively enrolled 18 consecutive athletes (12 males and six females, median age 27 years) with unilateral or bilateral chronic exertional compartment syndrome undergoing unilateral or bilateral minimally invasive fasciotomy. Clinical outcomes were assessed with Short-Form Health Survey-36 (SF-36) and European Quality of Life-5 Dimension (EQ-5D) scale. The ability to participate in sport before and after surgery and the time to return to training (RTT) and to sport (RTS) were recorded.

**Results:**

The median follow-up after surgery was 36 months. Both questionnaires showed a statistically significant improvement (*P* < 0.0001) after surgery. At the time of the latest follow-up, 17 of 18 patients (94 %) had returned to pre-injury or higher levels of sport. Only one patient (6 %) returned to sport at lower levels than those of pre-injury status. The median time to return to training and to return to sport was 8 and 13 weeks, respectively. No severe complications or recurrence of the symptoms were recorded.

**Conclusions:**

Minimally invasive fasciotomy is effective and safe for athletes suffering from unilateral or bilateral chronic exertional compartment syndrome of the anterior and lateral compartments of the leg with good results in the mid-term.

## Background

Chronic exertional compartment syndrome (CECS) of the leg is common in athletes. Repetitive movements may cause it, especially in running or endurance sport activities, impairing athletic performance [1, 2]. The intramuscular pressure may increase during exercise, leading to pain to the affected compartment, up to induce athletes to abandon any sport activity. Aetiologically, the muscle swelling, secondary to repetitive activities, and fascial tightness are mainly involved factors increasing the compartment pressure [3, 4]. Usually, pain disappears at rest, but the involved muscles may become ischemic [1, 5–8]. Therefore, surgical fasciotomy, which implies the decompression of the involved muscular compartments to decrease the pressure, is the treatment of choice [3, 9–14]. In athletes, the anterior, the lateral and the deep posterior compartments of the leg are commonly affected; the superficial posterior compartment is more rarely involved [2, 15].

Different surgical approaches may be undertaken, including open, endoscopic, and minimally invasive procedures [2, 10, 12, 15–20].

We report the mid-term results in a series of 18 consecutive athletes with chronic exertional compartment syndrome of the leg who had undergone minimally invasive fasciotomy of the leg.

## Methods

All procedures were performed after the local Ethical Committee of the Faculty of Medicine and Surgery of the University of Salerno (CESa 01252009/Rev2) had approved them and after patients had given their written informed consent. All the authors declare that the procedures followed were in accordance with the ethical standards of the responsible committee on human experimentation (institutional and national) and with the Helsinki Declaration of 1975, as revised in 2000 and 2008.

During the period from 2001 to 2007, we prospectively enrolled 18 consecutive athletes (12 males and six females) (median age 27 years; range from 18 to 35 years) who had undergone minimally invasive fasciotomy for management of leg pain secondary to chronic exertional compartment syndrome (Table 1). The condition was unilateral in nine of 18 patients, bilateral in nine patients. The sport activity was running in nine patients, ballet in four, soccer in two, badminton in one, and squash in two. The median duration of symptoms before surgery was 17 months (from 5 to 31 months).

**Table 1:** Demographic details and clinical outcomes of the enrolled athletes

Athlete	Gender	Sport	Affected side	Affected compartment	Pre-injury level of sport	Post-surgery level of sport	Scores			
							EQ-5D		SF-36	
							Pre-surgery	Post-surgery	Pre-surgery	Post-surgery
1	M	Soccer	R + L	Ant	Semi-professional	Semi-professional	51	90	52	86
2	M	Soccer	R	Ant	Semi-professional	Sunday league	55	92	51	90
3	M	Running	R + L	Ant + Lat	County	County	60	95	58	92
4	M	Running	L	Ant + Lat	County	County	58	90	54	91
5	M	Running	R + L	Ant	County	County	61	92	59	88
6	M	Running	R + L	Ant	Recreational	Recreational	59	91	51	94
7	M	Running	R + L	Ant + Lat	County	County	59	92	57	96
8	F	Running	R	Ant	County	County	53	90	58	97
9	M	Running	R + L	Ant	County	National	60	92	60	88
10	M	Running	R	Ant + Lat	County	County	64	91	52	90
11	M	Running	R + L	Ant	Recreational	Recreational	53	98	57	95
12	F	Ballet	R	Ant + Lat	Recreational	Recreational	62	94	51	85
13	F	Ballet	L	Ant + Lat	Recreational	Recreational	67	90	62	92
14	F	Ballet	R	Ant + Lat	Recreational	Recreational	66	89	55	91
15	F	Ballet	R + L	Ant	Recreational	Recreational	53	88	58	89
16	M	Badminton	R	Ant	Recreational	County	59	91	53	91
17	M	Squash	R + L	Ant + Lat	Recreational	Recreational	53	92	53	94
18	F	Squash	L	Ant	Recreational	Recreational	66	92	52	86

In this table, gender, type of sport performed, affected side, affected compartment, pre- and post-injury level of sport, and results of scores are reported. All results of EQ-5D and SF-36 scores are improved after surgery

*R* right, *L* left, *Ant* anterior, *Lat* lateral, *EQ-5D* European Quality of Life-5 Dimensions, *SF-36* Short-Form Health Survey-36

All patients were tertiary referrals to the senior author with the following diagnosis: CECS in six, persistent swelling in five, peroneal muscle strain in four, and shin pain in three. All athletes had been managed previously for a median period of 4 months (range from 1 to 6 months) with conservative therapies, which consisted in changing training modalities (shoes, stretching, shoe inserts), or application of local injection of local anesthetics at the site of maximal tenderness.

The diagnosis of CECS was made on history and clinical examination in all instances. All patients referred pain over the anterior/antero-lateral aspect of the leg after a well-defined period of exercise, generally 20 min (from 15 to 30). Symptoms always resolved after rest, within several minutes. At clinical examination, no patients complained of tenderness of the tibial shaft or surrounding soft tissues. Plain radiographs were undertaken to exclude a tibial stress fracture and other bony disorders.

The measures of the dynamic compartment pressure confirmed the diagnosis of CECS in all instances [21–24]. The intracompartmental pressure (ICP) of the anterior and lateral compartments was measured bilaterally, at rest and 1 min after exercise. Diagnostic criterion for CECS was a value of ICP >28 mmHg, 1 min after exercise [21]. In healthy legs, the median values of ICP were 13.6 mmHg (from 5.2 to 26.5) at rest and 17.5 mmHg (from 11 to 26.3), 1 min after exercise. In affected legs, the median values of ICP were 22.5 mmHg (from 6 to 51) at rest and 44 mmHg (from 28.4 to 122.7), 1 min after exercise.

### Clinical assessment

The Short-Form Healthy Survey-36 (SF-36) [25] and the European Quality of Life-5 Dimension (EQ-5D) [26] scores were administered to all patients at baseline and postoperatively, 6 weeks after surgery. These are self-administered rating systems assessing the overall health status, the physical and psychological function, with a final score ranging from 0 (lowest health status) to 100 (highest health status).

All patients were asked about participation to sport before surgery and at the last follow-up and the time they had taken to return to training (RTT) and to sport (RTS) [27].

At the last follow-up, the overall satisfaction of patients with surgery was evaluated according to a three-staged self-reported scoring system: none was a condition of minimal or no relief, moderate was significant relief, and good was return to full activity with minimal or no symptoms.

### Surgical technique

The senior author performed all procedures as day cases. With the patients in supine position and the tourniquet inflated to the tight, the skin was prepared in the usual fashion, and sterile drapes were applied.

**Fig. 1:**
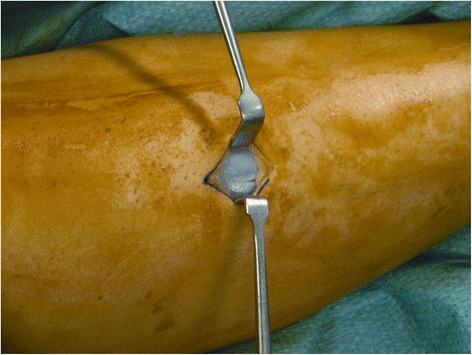
Retraction of subcutaneous tissues and visualization of the deep fascia

**Fig. 2:**
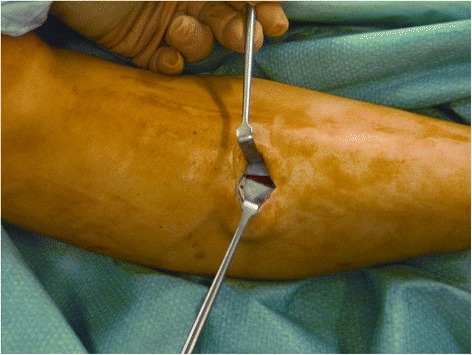
Incision of the deep fascia

**Fig. 3:**
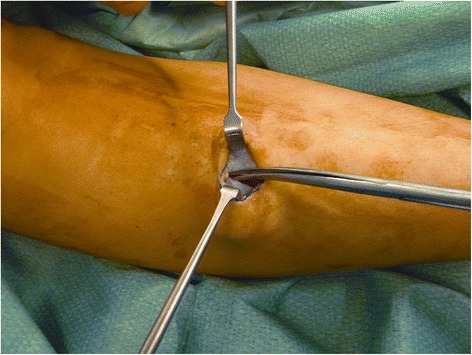
Cranial division of the anterior and lateral compartment fascia

**Fig. 4:**
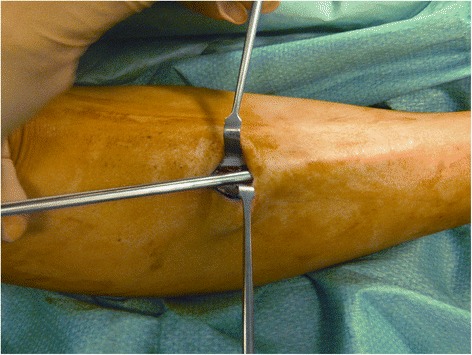
Caudal division of the anterior and lateral compartment fascia

A 2.5-cm vertical skin incision was performed over the anterior compartment, at the middle third of the leg, 1.0 cm lateral to the tibial crest. Once the fascia was identified, the skin was retracted, and subcutaneous tissues and layers down to the level of the fascia were carefully dissected with gloved fingers, proximally and distally. If required, to uncover the fascia, the blunt subcutaneous dissection was completed by pushing the closed scissors proximally and distally. In this way, when the deep fascia could be clearly visualized, it was incised with a knife, at an average distance of 1.0 cm from the intermuscular septum (Fig. 1). Under direct vision, the fascia of the anterior compartment was divided proximally and distally, with scissors (Figs. 2, 3, and 4). In the same way, when the lateral compartment was also affected, through the same incision, the skin was retracted posteriorly to expose the fascia of the lateral compartment, which was cut. The incision was closed with subcuticular Biosyn suture 3.0 (Tyco Healthcare, Cork, Ireland); steri-strips (3M Health Care, St Paul, MN, USA) were applied for the stab incisions. A Mepore dressing (Molnlycke Health Care, Gothenburg, Sweden) was applied.

Postoperatively, patients were discharged the day of surgery. They were allowed to weight bear as comfortable with elbow crutches and to move the ankle and knee. Patients used crutches for 7 to 10 days after the operation. At 2 weeks, the wounds were inspected, and rehabilitation was started, with gradual return to walking, jogging, and agility training. Competitive training was allowed at 4 weeks, at least; competitive sport was recommended not before than 8 weeks.

Clinical follow-ups were set at 2 and 6 weeks and 3, 6, and 12 months after surgery. Then, patients were examined once per year for a maximum of 4 years. The last follow-up was at a median time of 36 months (range from 26 to 50 months) after surgery.

### Data collection and statistical analysis

Data were recorded in a computer database and analysed using SPSS for Mac (version 16.0). Demographic data, history, physical examination findings, and values of compartment pressure were recorded. Descriptive statistics was calculated. Wilcoxon signed-rank test was used to compare preoperative and postoperative EQ-5D and SF-36 scores. Pearson’s chi-squared test with the Yates correction was used to assess the return to sport activity rate. Nonparametric statistical tests that were used as values were not found to be normally distributed. A *P* value of <0.05 was considered significant.

## Results

At the last follow-up, the median value of EQ-5D was increased from 59 (range from 51 to 67) at baseline to 92 (range from 88 to 98) (*P* < 0.0001); the median SF-36 from 55 (range from 51 to 62) at baseline to 91 (range from 86 to 97) (*P* < 0.0001). Specifically, the median value of the mental component of SF-36 was 54 (range from 49 to 60) preoperatively and 92 (range from 86 to 97) postoperatively (*P* < 0.0001); the median value of the physical component of SF-36 was 56 (range from 52 to 62) preoperatively and 90 (range from 84 to 95) postoperatively (*P* < 0.0001).

Before surgery, 12 of 18 patients were unable to practise any sport activity, the remaining six patients could practise sport activity at lower levels. At the last follow-up, 15 of 18 patients had returned to pre-injury levels of sport, two had gained higher levels of competition compared to the pre-injury status, one had returned to sport at lower level. After surgery, there was a statistically significant increase of the number of patients able to perform sport at pre-injury or higher level (from 0 to 94 %; *P* < 0.0001), and a statistically significant decrease of the number of patients fully unable to practise any sport (from 67 to 0 %; *P* < 0.0001) and those able to do sport at reduced levels (from 33 to 6 %; *P* < 0.0001). The median time to return to competitive training was 8 weeks (range from 4 to 12); the median time to return to competitive sport was 13 weeks (range from 9 to 18). At the last follow-up, the overall satisfaction for the procedure was graded as good in 17 of 18 patients, moderate in one patient.

Of 27 fasciotomies performed (nine bilateral), we recorded two superficial wound infections, resolved with oral antibiotics, one hypertrophic scar, and one skin stretching around the scar. Numbness around the wound developed in one patient, and spontaneously resolved within 8 weeks after the index procedure. One patient complained of weakness in ankle dorsiflexion. This event was managed with targeted physiotherapy, until the recovery was complete. No patients had recurrence of symptoms.

## Discussion

The main finding of the present study is that this minimally invasive fasciotomy through a single incision is safe and effective for management of chronic exertional compartment syndrome of the leg, allowing patients to return quickly to sport activities, with high overall satisfaction in the mid-term. The efficacy of fasciotomy as standard management for patients with CECS of the leg is well known [3, 4, 9, 12, 13, 15, 17, 19]. In our series, we used a single incision minimally invasive fasciotomy in patients with anterior or antero-lateral CECS of the leg. At a median follow-up of 3 years, outcomes were surprisingly good compared to the baseline and stable.

Fascial release may be performed using one or two incisions [10, 12, 15, 16, 18–20, 28, 29], three or more incisions in patients with long legs [3]. When performed endoscopically, the fascia may be visualized directly, using single or two incisions [30–32]. Nevertheless, the problem may recur, mostly if the release is incomplete. Some complications may also occur after fasciotomy: infections, haematoma, injury to the nerves and vessels, scars, fascial adhesions, swelling, lymphocele, and haemorrhage of the leg [3, 12, 15, 17]. Specifically, as shown in cadavers, the peroneal nerve may be injured at the junction between the middle and the distal third of the calf, 10 to 12 cm proximally to the lateral malleolus, regardless of the technique used [30]. However, the risk of injury to the peroneal nerve is lower when undertaking an endoscopically assisted fascial release of the anterior and the lateral compartments through a single incision. In the short term, the overall rate of complications is 22 %. In our series, none of the patients experienced severe complications or recurrence of symptoms. Only one patient presented weak dorsiflexion of the ankle that regressed after tailored physiotherapy [20, 33], and 2 of 11 patients who had undergone bilateral fasciotomy complained of leg weakness for an average of 3 months after surgery.

Our results were surprisingly encouraging, probably because all patients had anterior or antero-lateral CECS, which are reported to present better outcomes [4, 12, 13, 15, 17, 31] compared to compartment syndromes of the posterior compartment [1, 3, 9, 10, 15, 17, 34]. Mouhsine et al. [33] reported the long-term results of 18 athletes with anterior and/or lateral CECS of the leg, in whom the release of the fascia had been made through two minimal incisions, without tourniquet. The fact that patients had returned to sport activities at an average of 25 days after the index procedure, and all at the pre-injury level of activity, confirms that the double incision technique allows to decompress completely the anterior and lateral compartments, without major risks [12]. We also reported a good result, using a single incision. Our technique has some features in common with that performed by Mouhsine et al. [33]. First of all, the release of the skin and subcutaneous tissues allows to decompress the compartments under direct control. We also have used the gloved finger to perform a blunt dissection. The single incision technique may appear to be more technically demanding, but it allows complete decompression of the compartment by advancing the scissors subcutaneously, proximally, and distally. A small skin incision could not allow to release completely the fascia, as the wound edges can hinder the blades of the scissors. For this reason, we use long scissors, cutting the fascia at a remarkably long distance from the skin incision. Moreover, the fact that the branches of the scissors have not to be widely opened, it prevents any potential damage to the overlying skin. Finally, the surgeon may appreciate the consistence and resistance of the fascia released during the procedure, having care that the tip of the scissors is open. This makes it possible to assume that the fasciotomy is complete when the resistance cannot be appreciated anymore. We encourage early mobilization and weight-bearing to prevent scarring and adhesions [9, 12, 34], even though most patients may not weight bear immediately after the operation because of the pain. Thus, we recommend patients to weight bear as they are comfortable.

In the study by Mouhsine et al. [33], symptoms persisted less than 7 months; in our patients, the duration of symptoms was longer, probably in relation to the fact that our unit care is a tertiary referral centre. We are aware that a prolonged duration of symptoms (>12 months) from the first occurrence is a negative prognostic factor [33], but this was not observed in our study.

We well acknowledge that there are many procedures to perform fasciotomy but, to the best of our knowledge, this is the first study to describe a minimally invasive fasciotomy through a single minimal incision of both anterior and lateral compartments. Strengths of the study are its prospective nature, the fact that a single fully trained surgeon performed all the operations without any surgical-associated procedure, the patients were included according to strict selection criteria, and no patient was lost to the follow-up. Moreover, an independent reviewer not involved at surgery assessed the outcomes at the follow-ups. A critical point is that the postoperative program used in the study could not be the most appropriate, but it was uniform throughout the study. In spite of this, we now point that, ideally, the rehabilitation should be standardized in all the studies.

A major limitation of the study is the lack of a control group of patients, making it impossible to prove whether this procedure is superior to the previous techniques used for management of anterior and/or lateral CECS. We are aware that our evidence is not strong as that of randomized controlled trials but, in referral to the low frequency of this injury, these latter studies would likely be too long and costly to be performed. Nevertheless, given the results, our approach should be taken into account as management of these patients. Another limitation is represented by the outcome measures used. We acknowledge that the SF-36 and EQ-5D scores are generic tools of assessment of the health status, and that a disease-specific questionnaire should be used, but no scoring systems specific for CECS have been described yet.

## Conclusions

In conclusion, although this approach could appear technically demanding, it is safe and allows most of patients to recover completely and return to pre-injury sport levels of activity, with good overall satisfaction and physical function in the mid-term. Therefore, it may be considered as an alternative to traditional open surgery.

### Consent to publish

We have obtained consent to publish from the participants.

## Abbreviations

CECS: chronic exertional compartment syndrome
EQ-5D: European Quality of Life-5 Dimensions
ICP: intracompartmental pressure
RTS: return to sport
RTT: return to training
SF-36: Short-Form Health Survey-36
